# Three-Dimensional Kinematics during Shoulder Scaption in Asymptomatic and Symptomatic Subjects by Inertial Sensors: A Cross-Sectional Study

**DOI:** 10.3390/s22083081

**Published:** 2022-04-17

**Authors:** Cristina Roldán-Jiménez, Antonio I. Cuesta-Vargas, Jaime Martín-Martín

**Affiliations:** 1 Biomedical Research Institute of Málaga (IBIMA), 29010 Málaga, Spain; cristina.roldan005@gmail.com (C.R.-J.); jaimemartinmartin@uma.es (J.M.-M.); 2 Department of Physiotherapy, University of Málaga, 29071 Málaga, Spain; 3 Faculty of Health Science, School of Clinical Science, Queensland University Technology, Brisbane 4000, Australia; 4 Legal and Forensic Medicine Area, Department of Human Anatomy, Legal Medicine and History of Science, Faculty of Medicine, University of Málaga, 29071 Málaga, Spain

**Keywords:** shoulder, functional assessment, kinematics, inertial sensors

## Abstract

Shoulder kinematics is a measure of interest in the clinical setting for diagnosis, evaluating treatment, and quantifying possible changes. The aim was to compare shoulder scaption kinematics between symptomatic and asymptomatic subjects by inertial sensors. Methods: Scaption kinematics of 27 subjects with shoulder symptomatology and 16 asymptomatic subjects were evaluated using four inertial sensors placed on the humerus, scapula, forearm, and sternum. Mobility, velocity, and acceleration were obtained from each sensor and the vector norm was calculated from the three spatial axis (*x*,*y*,Z). Shoulder function was measured by Upper Limb Functional Index and Disabilities of the Arm, Shoulder, and Hand questionnaires. One way ANOVA was calculated to test differences between the two groups. Effect size was calculated by Cohen’s d with 95% coefficient Intervals. Pearson’s correlation analysis was performed between the vector norms humerus and scapula kinematics against DASH and ULFI results in symptomatic subjects. Results: The asymptomatic group showed higher kinematic values, especially in the humerus and forearm. Symptomatic subjects showed significantly lower values of mobility for scapular protraction-retraction (Cohen’s d 2.654 (1.819–3.489) and anteriorisation-posteriorisation (Cohen’s d 1.195 (0.527–1.863). Values were also lower in symptomatic subjects for velocity in all scapular planes of motion. Negative correlation showed that subjects with higher scores in ULFI or DASH had lower kinematics values. Conclusion: Asymptomatic subjects tend to present greater kinematics in terms of mobility, velocity, and linear acceleration of the upper limb, and lower humerus and scapula kinematics in symptomatic subjects is associated with lower levels of function.

## 1. Introduction

The shoulder joint complex consists of four anatomical joints and another virtual one (subacromial) that gives it excellent mobility [1]. Broad mobility is detrimental to joint stability [2], making the shoulder prone to several dysfunctions and pathologies [3]. Scapulo-humeral rhythm (SHR) or glenohumeral rhythm was defined by Codman in 1934 [4]. This coordinate movement between the scapula and humerus is needed for efficient arm movement, the alignment of the glenohumeral joint, and to maximize stability [5]. Different shoulder injuries could produce an alteration of movement [6,7].

Mobility is limited in subjects with chronic shoulder pain, and activities of daily living are affected [8]. Shoulder kinematics is, therefore, a measure of interest in the clinical setting [9], since this aspect is important for diagnosis, evaluating treatment and quantifying possible changes [10]. In the kinematic field, among the most used devices in recent years has been inertial sensors, since their small size and portability have overcome the gap between laboratory systems and those used in the clinic. Moreover, they have shown to represent a valid, reliable system for movement analysis [11]. In this sense, the application and development of sensors to monitor physiological and kinematics variablesare under development. However, the use of the inertial measurement unit (IMU) and other kinds of sensors requires technical training of the clinician to know the data and to be able to apply them properly in their professional practice [12].

Shoulder kinematics measured by inertial sensors has been extensively studied. There is now evidence of the operational feasibility of these devices in various clinical applications [13]. Their reliability and validity have been reviewed compared to optoelectric systems in the upper limb, trunk, or lower limb with high validity [11,14], and several protocols have been developed for analyzing upper limb movements [15], the scapulothoracic, thoracohumeral, and elbow joints [16], scapula, and SHR [17]. However, once protocols and validity have been established, there is a need for further research in order to provide descriptive values that provide information in the clinical setting. Furthermore, although kinematics covers mobility, velocity, and acceleration [18], previous research has focused mostly on mobility [19].

One of the planes of motion of most interest is ‘scapular plane elevation’, also called ‘scaption’. Being considered one of the main exercises to stabilize the shoulder joint, scaption is described with the patient’s arm flexed by 30° with the thumbs positioned upward [20]; the plane of the scapula and humerus are aligned, which makes SHR and kinematic measures easier. Given that scaption refers to functional movement patterns involved in shoulder biomechanics [21], it is an ideal exercise for muscle strengthening in open chain elevation and beyond the shoulder [19]. In addition, scaption movement decreases the risk of injury by placing the greater tubercle’s apex under the coracoacromial arch’s high point [3]. Therefore, scaption is considered the most efficient movement plane for exercises in the clinical field [22].

Due to the importance of shoulder scaption and affected kinematics in shoulder injuries, this study hypothesizes that there may be differences in mobility, velocity, and acceleration between healthy and symptomatic subjects. Hence, this study aimed to compare upper limb kinematics during shoulder scaption between symptomatic subjects suffering from shoulder injury and asymptomatic controls using inertial sensors in terms of mobility, velocity, and acceleration.

## 2. Materials and Methods

### 2.1. Participants

This cross-sectional study recruited adult subjects suffering from shoulder injury (symptomatic group) and adults without shoulder pain (asymptomatic group). The subjects met all inclusion and exclusion criteria and gave informed consent to participate in the project. The study had ethics approval from the Ethics Committee of the Research Commission of the Faculty of Health Sciences of the University of Malaga. The principles of the Declaration of Helsinki were respected.

Patients were recruited from a specialized orthopedics clinic where they had been previously diagnosed by magnetic resonance imaging [23] and were on the waiting list for surgery. Subjects were included if they wanted to participate in the study and aged between 18 and 75 years, as older participants could offer confusing results in the reading of the inertial sensors due tremors or small motor disorders. Subjects were excluded if they had any history of surgical intervention, fracture, or previous bone/joint history in the upper limbs, or if they suffered from any systemic disease related to the shoulder joint. Subjects with mental impairments which made it impossible to perform the tasks were excluded. Athletes or professional player were not included to improve the homogeneity of the sample [24]. Asymptomatic subjects were excluded if they had experienced any shoulder pain in the last three months, based on the Disabilities of the Arm, Shoulder and Hand (DASH) [25] or Upper Limb Functional Index (ULFI) [26], as they had to have 0 punctuation in both questionnaires. They were also excluded if they presented positive Neer [27] or Hawkins tests [28].

Twenty-seven patients (10 men and 17 women) were recruited. Affected shoulders were measured (8 left arms and 19 right arms). This group of participants presents the following diagnoses: 15 were suffering from rotator cuff tears (RCT), 7 were suffering from impingement syndrome; 3 from supraspinatus tendinopathy; 1 from shoulder instability; and 1 from slap lesion. A total of 32 right shoulders and 11 left shoulders were measured. Hence, 5 out of 27 measures were carried out in the shoulder from the non-dominant hemibody. In the asymptomatic group, 3 left arms and 13 right arms were measured, in order to have measures from right and left sides, as in the symptomatic group. Further, 3 out of 16 measures were carried out in the non-dominant shoulder.

### 2.2. Equipment and Outcome Measures

Four inertial sensors; InertiaCube3 (Intersense Inc., Billerica, MA, USA) and the InertiaCube3™ software, Intersense Server, were used to register kinematic scaption movement with a sampling frequency of 1000 Hz. Intersense sensors have been validated in previous studies for human motion analysis compared to other reference devices [29,30,31]. Each inertial sensor has an accelerometer, a gyroscope, and a magnetometer inside. Nine inertial variables were obtained: mobility angle (°), angular velocity (°/s), and linear acceleration (m/s^2^), for each of three spatial axes: *x*, *y*, and *z* of the inertial sensor (Table 1). Kinematic data were recorded by kinematic Intersense Server Software, and were subsequently transferred to a database in Microsoft Excel 2007. Based on RAW data, the highest point that the patient reached in scaption movement during arm elevation recorded in the sensor placed on the humerus was used as a cut-off point, and the corresponding angles obtained at the lowest point reached by each sensor were subtracted to calculate angular mobility. No signal filter was applied to obtain the largest amount of data. Angular mobility was expressed in degrees (°). The minimum peaks obtained by each sensor and corporal segment were subtracted from the maximum peaks in order to calculate angular velocity (°/s) and linear acceleration (m/s^2^).

Sensors were placed following the protocol established by Cutti et al. [16]. This protocol is valid, reliable, and accurate. It is also highly correlated to the gold standard optoelectronic system [16]. Sensor placement was in the hemi body of the affected shoulder of each patient as follows:

(1) the middle third of the humerus, a little posterior with the Z axis pointing against the body;
(2) on the medial third of the scapular upper spine with the *x* axis in alignment with the cranial edge of the scapular spine;
(3) on the sternum with the Z axis pointing against the body; and
(4) on the distal surface of the ulna and radius with the Z axis pointing away from the wrist.

The placement has been previously validated compared to other positions [32]. Table 1 shows the equivalence between the axes in each sensor and the different axes and planes of anatomical movement during shoulder motion following the presented protocol.

Descriptive and anthropometric variables related to age, gender, weight, and height were obtained. Information regarding shoulder disability was measured by two questionnaires, namely the Spanish DASH [25] and ULFI [26] version. The DASH questionnaire is a standardised measure of upper limb functional status and symptoms [33]. It has been shown to be a valid, reliable questionnaire for patient populations suffering from several upper limb disorders [34]. ULFI has also shown to have strong psychometric properties for reliability and validity [35]. Each participant filled both questionnaires and transferred to a 100-point scale.

**Figure 1:**
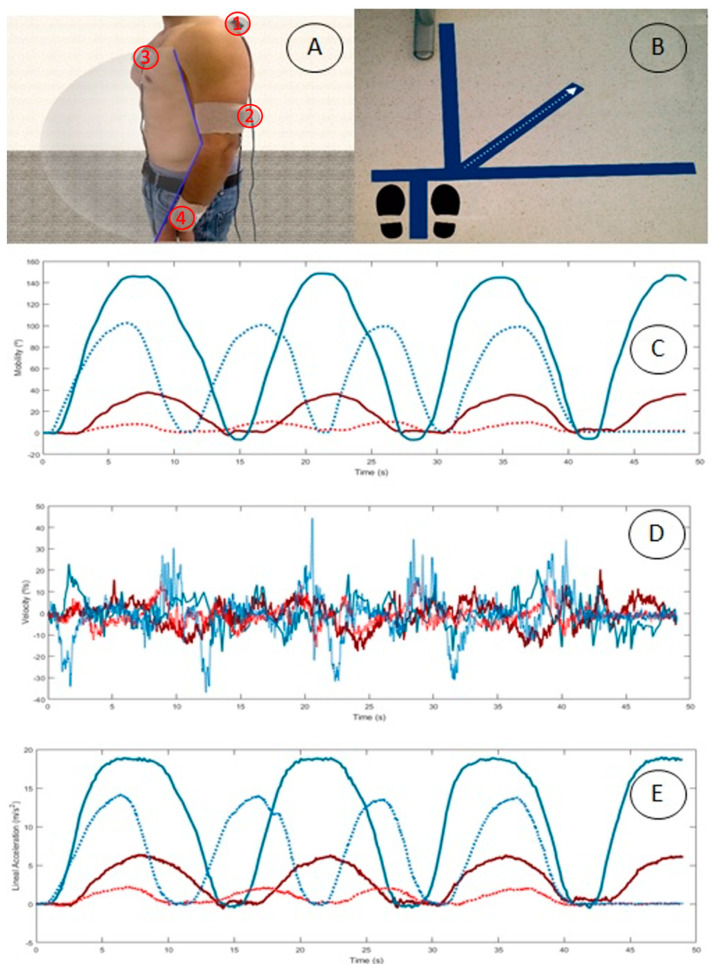
(**A**) Inertial sensors placement in the scapula (1), humerus (2), sternum (3) and forearm (4). (**B**) Mark placed on the floor to indicate frontal, lateral and scapular plane, as an intermediate line. (**C**) Example of SHR (°) during scaption. Blue represent humerus AB-AD. Red represents scapular PR-RE in asymptomatic control (continuous line) and pathological subject (discontinuous line). (**D**) Example of SHR (°/s) during scaption. Blue represent humerus AB-AD. Red represents scapular PR-RE in asymptomatic control (continuous line) and pathological subject (discontinuous line). (**E**) Example of SHR (m/s2) during scaption. Blue represent humerus AB-AD. Red represents scapular PR-RE in asymptomatic control (continuous line) and pathological subject (discontinuous line). SHR = Scapulo-humeral rhythm.

Before attaching sensors, body surfaces were cleaned with alcohol for better adhesion to the skin. Inertial sensors were reset to zero using the Intersense Server Software on a vertical flat surface, as suggested by the manufacturer. The sensors were attached using double-sided adhesive tape to ensure fixation. A cohesive elastic bandage was used on cylindrical body segments (arms and forearm), and an adhesive bandage was used on flat areas (sternum and scapula) in order to reduce sensor movements due to muscle fat (see Figure 1A).

### 2.3. Procedure

After recruitment, participants attended the study in the Human Movement Laboratory, Faculty of Health Science of Malaga University. Participants were first asked to fill in the questionnaires. Sensors were then fitted, and they were asked to perform shoulder scaption. Participants stood on a mark placed on the floor for this purpose. Hence, 3-line marks were made to indicate the frontal, lateral, and scapular plane, as an intermediate line (see Figure 1B). Participants were placed standing in the neutral position to perform shoulder elevation in the scapular plane, keeping the elbow extended, the wrist in the neutral position, and the palm toward the body’s midline at the beginning and end of the scaption. Subjects were told to perform shoulder scaption to the highest position they could reach. A preselected speed was not given so as not to influence velocity and acceleration variables.

Once the procedure was explained and it was confirmed that the participant had understood the action to be carried out, participants were told to perform four repetitions for the record to save the second one. The beginning and end of each task were indicated verbally by the researcher.

### 2.4. Data Analysis

Priori sample size was calculated for an α error of 0.05, a statistical power of 0.8 and an effect size of 0.98. Calculation made by G*Power (Version 3.1.9.2) showed a total sample of 14 subjects for one-way ANOVA test between 2 groups based on kinematic data from shoulder scaption. The data used for the sample size estimation were an average maximum flexion of 153.9 degrees (SD 8.8) for control subjects and 142.8 degrees (SD 13.2) for subjects with impingement in scaption movement [36]. SPSS v22.0 was used for all statistical computations. Descriptive statistics (mean, standard deviation) were calculated for all variables using standard procedures. An analysis of variance, one-way ANOVA, with F and *p* values was calculated in inertial variables (mobility, velocity and linear acceleration) to test differences between the two groups for each of the measured body segments (humerus, forearm, scapula, and thorax). Effect size was calculated by Cohen’s d with 95% coefficient intervals (CI 95%) in order to complement ANOVA. ANOVA models were performed on the values obtained in the humerus, scapula, arm, and forearm to observe the differences between symptomatic and asymptomatic subjects in mobility, speed and linear acceleration outcomes. Lower values were represented by Cohen’s d < 0.2, whereas Cohen’s d < 0.5 indicated medium values. Cohen’s d > 0.8 was considered a high value [37]. The Kolmogorov–Smirnov test showed normal data distribution (*p* > 0.05). For all statistical comparisons, the α level was set at 0.05. Pearson’s correlation analysis was performed between the vector norms variables of mobility, velocity and acceleration of the humerus and scapula against DASH and ULFI results in symptomatic subjects.

## 3. Results

The total sample (*n* = 43) was composed of subjects of similar ages, with a mean of 55 in the asymptomatic and 52 years in the symptomatic group, respectively. No statistical differences were found in age, weight, height, and BMI. The symptomatic group had (70.07 ± 24.51) and (63.19 ± 20.38) points in ULFI and DASH questionnaires, respectively.

Figure 1C shows examples of the SHR mobility pattern represented by humeral abduction-adduction (AB-AD) and scapular protraction-retraction (PR-RE) in a patient and an asymptomatic subject during shoulder scaption. Figure 1D represents velocity and Figure 1E represents linear acceleration.

In all sensors, descriptive values from the symptomatic and asymptomatic groups were obtained in terms of mobility, velocity, and acceleration in all sensors (Table 2, Table 3 and Table 4). As for humeral kinematics, the asymptomatic group showed greater mobility and acceleration. The highest significant mobility and acceleration were found in the AB-AD movement, and the highest significant velocity was found in the flexion-extension (FL-EX) component in both groups. Although the values were higher for velocity for AB-AD and internal-external axial rotation (IN-EX) humeral movements in the symptomatic group, these differences were not significant (Table 2). Regarding the scapula, patients showed significantly lower mobility values for PR-RE and anterior-posterior tiling (AN-PO). In the symptomatic group, values were also lower for velocity and acceleration, with significant differences for velocity in all planes of motion (Table 2).

Regarding the forearm sensor, asymptomatic subjects presented higher FL-EX and pronation-supination (PR-SU) for all variables, representing a greater elbow component. However, these differences were only significant in mobility and FL-EX acceleration. The same was observed for the sternum sensor, with these higher values being significant, except for velocity in axial rotation (Table 3 and Table 4). Correlation analysis examined whether the vector norm of mobility, velocity, and acceleration was associated with DASH and ULFI, focusing on symptomatic cases (Table 5). An inverse correlation was shown. Higher levels of ULFI and DASH imply a higher degree of dysfunction. Thus, subjects with higher scores in ULFI or DASH have lower mobility, speed, or acceleration, observed in negative values in terms of correlation. The highest values of correlation and greater statistical significance were observed in the mobility of the humerus with the DASH and ULFI. Thus, higher values in the DASH and ULFI tests would imply a decrease in the mobility of the humerus.

## 4. Discussion

The present study compared upper limb kinematics between asymptomatic subjects and symptomatic patients in terms of mobility, velocity, and acceleration, using four inertial sensors placed on the humerus, scapula, forearm, and sternum during scaption motion. All variables tended to be greater in the asymptomatic group, and significant differences depended on the plane of motion and the kinematic variable analyzed.

As far as the authors are aware, this is the first study that offers results regarding velocity and acceleration during shoulder scaption, providing information about the quality and quantity of motion [11]. Although kinematics description includes mobility, velocity, and acceleration [18], most research has mainly studied shoulder joint angles [19]. Indeed, beyond many studies assessing shoulder mobility, alterations in the presence of injury remain controversial, and there is no clear relationship between dyskinesia and a specific pathology [38].

As for humeral kinematics, differences were found in AB-AD in mobility and acceleration variables. However, in the velocity variable, FL-EX was the plane of motion that showed differences, with lower values in the symptomatic group. Significant differences in both AB-AD and FL-EX planes of motion would be explained by shoulder scaption itself, as it implies arm elevation with a flexion component [21]. The lower values obtained in the asymptomatic group concur with those obtained by fluoroscopic images during shoulder flexion in damaged shoulders, which was limited to 59° (±25°) [39]. However, results are not comparable due to differences in methodology, the plane of motion analyzed, and age (subjects aged on average 74 years). Humerus elevation during scaption was recently studied by Pascual et al. [40] using the Kinescan/IBV stereophotogrammetry system. Their study included subjects suffering from rotator cuff tendinopathy (aged 46.89 (±10.69) years) and full-thickness RCT (aged 57 (±7.07) years), obtaining a value of 121.14° (±6.51°) and 100.50° (±9.46°), respectively. Differences found between shoulder damage were significant [40]. In both cases, maximum humeral elevation was higher than the sample of the present group (88.80° in AB-AD plane and 38.62 in FL-EX plane). Compared to the present sample, differences could also be explained by the different etiologies of shoulder damage. Nevertheless, results are not comparable, as in the study compared above, subjects held different weights while performing shoulder scaption. Furthermore, subjects were sitting, which may influence kinematics [41]. Pascual et al. [40] also included maximum humeral velocity while holding 250 g weight, which was 96.79°/s and 45.38°/s for the rotator cuff tendinopathy group and full-thickness RCT group, respectively. Values were lower when performing scaption with kg: 76.04°/s and 29.22°/s, respectively. In our study, maximum humerus velocity during scaption was 90.22°/s for AB-AD, 106.09°/s in FL-EX plane and 71.58°/s in IN-EX, with all of them presenting significant differences to the asymptomatic group. Humerus velocity, therefore, seems to be a key variable for inclusion in shoulder assessment.

As for scapular kinematics, patients showed significantly lower values for mobility for PR-RE and AN-PO, with ME-LA mobility being higher in the asymptomatic group but without significant differences. Increased ME-LA mobility is in line with a study that found an increased scapular ME-LA in patients with osteoarthritis and frozen shoulder as a compensatory pattern in symptomatic shoulders compared with the contralateral one [42]. This compensatory pattern has also been reported among healthy participants with experimental pain induction [43]. However, our finding contrasts with another study that found less ME-LA in some planes of arm elevation including scaption [44]. Moreover, PRE-RE and AN-PO were the movements that showed significant differences in the mobility variable. This lack of agreement in findings when studying scapular movement has been discussed recently in a systematic review, which did not find a consensus regarding scapular kinematic differences in the presence of shoulder pathology [38]. However, significant differences were obtained in all planes of scapular velocity. This reinforces the importance of measuring this variable to report how this motion is performed [11]. For example, this study found no differences in scapular ME-LA mobility, although the symptomatic group presented significantly lower values of ME-LA velocity (28.37°/s) than the asymptomatic group (44.06°/s). In any case, the results are not comparable because previous research is limited to mobility and does not include velocity. There was no significant difference concerning the acceleration variable. In light of these results, velocity seems to be a key variable when studying scapular kinematics during scaption motion.

As for sternum, the symptomatic group showed significantly lower values in all variables except axial rotation velocity. These lower values could be explained by the lower range of motion performed during shoulder scaption in the humerus and scapula. According to previous research, during unilateral arm movements, the trunk tends to perform a lateral and axial flexion to reach the full range of motion [1], as well as an extension [45]. The symptomatic group performed a mean of 88.80° humerus abductions compared to the mean of 138.98° performed by the asymptomatic group. The wider the range of motion, the larger the trunk compensation to reach full motion. This trunk compensation strategy should be considered as part of shoulder assessment [46].

Overall, the symptomatic group showed higher standard deviations for all variables. This tallies with previous research indicating that the clinical expression of shoulder injuries is highly variable [47,48,49]. Although this symptomatic group presented alterations from different etiologies, previous research focusing on specific damage, such as full-thickness RCT, has also shown functional variability under the same diagnosis [48]. Indeed, current research is moving toward diagnoses based on kinematics instead of anatomic diagnosis [50]. Therefore, measuring shoulder kinematics as part of shoulder assessment is vital, as current clinical shoulder measurements lack reliability [51]. Static measurements [52], scapular position and mobility tests [53], or clinical tests [54] could be complemented by three-dimensional kinematics, including variables such as scapular velocity, which is difficult for clinicians to assess objectively. In addition, the negative values found in the Pearson correlation (Table 5) show that subjects with higher scores in ULFI or DASH presented lower scapula or humerus kinematics. Thus, patient-reported outcomes such as these questionnaires are very useful for complementing shoulder assessment. Moreover, inertial sensors for measuring kinematic patterns provided traces during scaption performance (Figure 1). This real-time kinematic biofeedback could be implemented as part of treatment [55]. The development of the new textile sensors represents a great advance for obtaining accurate data, facilitating patients’ physiological and biomechanical analysis. Currently, there are many challenges and opportunities regarding the development of materials that improve the performance and detection of variables for better practical application. Elements such as real-time biofeedback used as a biomarker could be relevant for medical care. This would improve continuous monitoring of chronic diseases [12].

One of the limitations of the present study is the inclusion of shoulder lesions of different etiologies in the symptomatic group, as kinematic variables from scaption motion assessment seem to depend on the etiology of the shoulder injury [40]. Another limitation is that pain was not measured, which has been shown to increase movement variability [56]. Gender could be another limitation. As the literature correlates female gender with lower shoulder function in patients affected by RCT [57,58], there may be differences between men and women, but sample size and balance did not allow this analysis with full guarantees. Finally, this study used inertial sensors. These apparatuses are non-invasive, reliable, and accurate tools used to analyze motion [11], but one of their limitations is that placing sensors over the skin can create soft tissue artefacts [15], especially if subjects with high BMI are measured. Although the results provided by the IMUS are validated at a technical level, there are still limitations in their application in healthcare practice.

## 5. Conclusions

The present study analyzed differences in shoulder kinematics during shoulder scaption between asymptomatic and symptomatic subjects in the humerus, scapula, forearm, and sternum. As highlighted, this study included velocity and acceleration variables. Although all variables tended to be greater in the asymptomatic group, differences depended on the plane of motion and the kinematic variable analyzed. Specifically, significant differences were found in all planes of scapular velocity, which seems to be a key variable in symptomatic shoulders. Lower humerus and scapula kinematics values in symptomatic subjects were associated with lower levels of function. The results reinforce using these devices to complement the clinical assessment, as it offers information about motion quality.

## Figures and Tables

**Table 1:** Equivalence of the yaw, pitch and roll with the movement that they represent.

Surface Placement	Humerus	Ulna and Radius	Scapula	Sternum
Anatomical Segment Represented Axis	Humerus	Forearm	Scapula	Thorax
X	IN-EX	PR-SU	AN-PO	Axial rotation
Y	AB-AD	FL-EX	PR-RE	Flexion and extension
Z	FL-EX	Carrying angle	ME-LA	Lateral rotation

AB–AD, abduction–adduction; AN–PO, anterior–posterior tiling; FL–EX, flexion–extension; IN–EX, axial rotation; ME–LA, medio-lateral rotation; PR–RE, protraction–retraction; PR–SU, pronation–supination.

**Table 2:** Humerus and Scapula kinematics variables mean (SD) in Asymptomatic subjects and Symptomatic and difference between groups.

		Mobility (°)				Velocity (°/s)				Linear Acceleration (m/s^2^)			
		Asymptomatic	Symptomatic	ANOVA (F,*p*)	Cohens’d (95% C.I)	Asymptomatic	Symptomatic	ANOVA (F,*p*)	Cohens’d (95% C.I)	Asymptomatic	Symptomatic	ANOVA (F,*p*)	Cohens’d (95% C.I)
HUMERUS	AB-AD	138.98 (13.55)	88.80 (38.92)	20.104 (<0.001)	1.556 (0.864–2.267)	84.78 (37.59)	90.22 (155.9)	0.015 (0.903)	−0.043 (−0.062–0.575)	17.90 (2.61)	12.28 (6.20)	9.676 (0.004)	1.084 (0.425–1.744)
	FL-EX	57.24 (27.08)	38.62 (38.03)	2.453 (0.126)	0.541 (−088–1.17)	181.76 (49.63)	106.09 (58.77)	15.676 (<0.0001)	1.316 (0.679–2.043)	10.90 (2.97)	8.73 (8.78)	0.741 (0.395)	0.307 (−0.321–0.922)
	IN-EX	60.29 (50.20)	41.55 (28.28)	2.186 (0.148)	0.496 (−0.131–1.123)	72.15 (31.66)	71.58 (63.01)	0.001, (0.976)	0.011 (−0.608–0.629)	5.23 (1.76)	5.71 (4.83)	0.120 (0.731)	−0.12 (−0.739–0.499)
SCAPULA	AN-PO	18.85 (10.17)	7.81 (8.66)	11.576 (0.002)	1.195 (0.527–1.863)	50.19 (13.89)	30.83 (26.49)	5.580 (0.024)	0.853 (0.209–1.497)	3.15 (1.12)	2.48 (3.74)	0.360 (0.552)	0.219 (−0.401–0.839)
	ME-LA	5.38 (3.69)	5.47 (4.01)	0.005 (0.946)	−0.023 (−0.641–0.595)	44.06 (13.61)	28.37 (19.22)	6.351 (0.017)	0.903 (0.256–1.55)	4.32 (2.44)	3.31 (3.27)	0.893 (0.351)	0.337 (−0.285–0.96)
	PR-RE	31.89 (8.63)	10.03 (8)	56.663 (<0.001)	2.654 (1.819–3.489)	48.99 (20.37)	27.33 (18.20)	10.468 (0.003)	1.139 (0.475–1.802)	5.85 (1.43)	4.50 (4.91)	0.867 (0.358)	0.337 (−0.285–0.96)

AB–AD: abduction–adduction; AN–PO: anterior–posterior tiling; FL–EX: flexion–extension; IN–EX: internal-external axial rotation.; ME–LA: medio-lateral rotation; PR–RE: protraction–retraction.

**Table 3:** Forearm kinematics variables mean (SD) in Asymptomatic subjects and Symptomatic and difference between groups.

	Mobility (°)				Velocity (°/s)					Linear Acceleration (m/s^2^)		
	Asymptomatic	Symptomatic	ANOVA (F,*p*)	Cohens’d (95% C.I)	Asymptomatic	Symptomatic	ANOVA (F,*p*)	Cohens’d (95% C.I)	Asymptomatic	Symptomatic	ANOVA (F,*p*)	Cohens’d (95% C.I)
Carrying angle	44.22 (47.59)	47.17 (25.45)	0.060 (0.809)	0.084 (−0.702–0.535)	173.03 (46.62)	112.41 (74.21)	6.638 (0.015)	0.926 (0.277–1.574)	11.64 (3.21)	7.70 (2.39)	17.214 (<0.001)	1.426 (0.738–2.114)
FL-EX	150.44 (10.95)	94.26 (36.35)	27.066 (<0.001)	1.892 (1.156–2.628)	116.58 (38.53)	94.38 (68.50)	1.079 (0.306)	0.374 (−0.249–0.998)	19.23 (3.07)	12.98 (5.72)	12.387 (0.001)	1.271 (0.596–1.945)
PR-SU	73.61 (62.62)	43.70 (31.11)	6.638 (0.015)	0.661 (0.027–1.295)	102.84 (26.10)	81.73 (36.70)	3.149 (0.085)	0.636 (0.003–1.268)	6.29 (1.70)	5.59 (1.73)	1.307 (0.261)	0.407 (−0.217–1.032)

FL–EX: flexion–extension; PR–SU: pronation–supination.

**Table 4:** Sternum kinematics variables mean (SD) in Asymptomatic subjects and Symptomatic and difference between groups.

	Mobility (°)				Velocity (°/s)				Linear Acceleration (m/s^2^)			
	Asymptomatic	Symptomatic	ANOVA (F,*p*)	Cohens’d (95% C.I)	Asymptomatic	Symptomatic	ANOVA (F,*p*)	Cohens’d (95% C.I)	Asymptomatic	Symptomatic	ANOVA (F,*p*)	Cohens’d (95% C.I)
Lateral rotation	15.72 (9.38)	6.25 (8.12)	10.285 (0.003)	1.101 (0.44–1.762)	24.05 (8.46)	14.58 (9.61)	8.852 (0.05)	1.029 (0.373–1.684)	2.01 (0.75)	1.32 (0.85)	5.952 (0.020)	0.847 (0.203–1.491)
Flexion and extension	4.71 (2.60)	1.29 (2.14)	18.438 (<0.001)	1.475 (0.782–2.167)	21.44 (5.59)	15.30 (10.72)	3.677 (0.63)	0.669 (0.034–1.303)	1.38 (0.657)	0.65 (0.37)	18.967 (<0.001)	1.476 (0.783–2.168)
Axial rotation	14.98 (6.34)	5.87 (7.01)	15.173 (<0.001)	1.345 (0.665–2.026)	19.43 (5.67)	11.55 (5.33)	17.604 (<0.001)	1.444 (0.754–2.134)	1.09 (0.34)	0.73 (0.38)	7.997 (0.008)	0.984 (0.332–1.636)

**Table 5:** Correlation Person Values for symptomatic subject.

	**Humerus Mobility**		**Scapula Mobility**		**Humerus Velocity**		**Scapula Velocity**		**Humerus Acceleration**		**Scapula Acceleration**	
	**R Value**	***p* Value**	**R Value**	***p* Value**	**R Value**	***p* Value**	**R Value**	***p* Value**	**R Value**	***p* Value**	**R Value**	***p* Value**
**ULFI100**	−0.690	˂0.005	−0.766	3.259	−0.327	0.095	−0.401	0.038	−0.493	0.009	−0.556	˂0.005
**DASH100**	−0.682	˂0.005	−0.845	3.030	−0.277	0.161	−0.393	0.042	−0.458	0.016	−0.487	0.010

## Data Availability

The data presented in this study are available on request from the corresponding author.
